# Artificial intelligence evaluation of COVID-19 restrictions and speech therapy effects on the autistic children’s behavior

**DOI:** 10.1038/s41598-022-25902-y

**Published:** 2023-03-15

**Authors:** Fereshteh Sabzevari, Omid Amelirad, Zohre Moradi, Mostafa Habibi

**Affiliations:** 1 School of Rehabilitation Science, Iran University of Medical Sciences, Tehran, Iran; 2 Faculty of Engineering and Technology, Sharif University of Technology, Tehran, Iran; 3 Department of Electrical Engineering, Faculty of Engineering and Technology, Imam Khomeini International University, Qazvin, 34149-16818 Iran; 4 Department of Biomaterials, Saveetha Dental College and Hospital, Saveetha Institute of Medical and Technical Sciences, Chennai, 600077 India; 5 Institute of Research and Development, Duy Tan University, Da Nang, 550000 Vietnam; 6 Faculty of Electrical and Electronic Engineering, Duy Tan University, Da Nang, 550000 Vietnam

**Keywords:** Electrical and electronic engineering, Disability, Human behaviour, Health policy, Health services, Public health, Quality of life, Therapeutics

## Abstract

In the present study, we aimed to quantify the effects of COVID-19 restrictions and speech treatment approaches during lockdowns on autistic children using CBCL and neuro-fuzzy artificial intelligence method. In this regard, a survey including CBCL questionnaire is prepared using online forms. In total, 87 children with diagnosed Autism spectrum disorders (ASD) participated in the survey. The influences of three treatment approaches of in-person, telehealth and public services along with no-treatment condition during lockdown were the main factors of the investigation. The main output factors were internalized and externalized problems in general and their eight subcategory syndromes. We examined the reports by parents/caregivers to find correlation between treatments and CBCL listed problems. Moreover, comparison of the eight syndromes rating scores from pre-lockdown to post-lockdown periods were performed. In addition, artificial intelligence method were engaged to find the influence of speech treatment during restrictions on the level of internalizing and externalizing problems. In this regard, a fully connected adaptive neuro fuzzy inference system is employed with type and duration of treatments as input and T-scores of the syndromes are the output of the network. The results indicate that restrictions alleviate externalizing problems while intensifying internalizing problems. In addition, it is concluded that in-person speech therapy is the most effective and satisfactory approach to deal with ASD children during stay-at-home periods.

## Introduction

During COVID-19 pandemic, there have been some non-expected effects on the society besides the direct health effects of the COVID-19. The lockdowns all over the world have altered the education process and in many regions it took months for adaption to the new conditions. The autistic children’s education and treatment have been also significantly affected during the lockdown period. In Iran, restrictions due to COVID-19 pandemic were initiated 1 month after official recognition of the first confirmed COVID-19 patient. In addition, the effects of the restrictions on the special groups of the society were unclear. The condition for children with autism spectrum disorders (ASD) who receive regular treatments initiated some concerns. On the other hand, the condition for parents escalated since they had to care their children mostly on their own.

One of the main characteristics of ASD is flexibility difficulties, and therefore, the difficulty of adapting to new circumstances, such as the lockdown, causes some concerns. Moreover, increase of the intensity and frequency of behavioral problems in autistic children was frequently reported. In addition, prior diagnosed behavior difficulties predicted inferior outcomes of behavioral problems during lockdown periods. Change in daily routines of children and their parents, having to interact with more people during daily life in home, non-professional efforts from home members to deal with autistic children’ problems and lack of access to therapists are among main factors intensifying problems in this group of children. Alonso-Esteban et al.^1^ systematically reviewed the articles published on the effect of the COVID-19 restriction on autistic people and their caregivers. When addressing parents/caregivers, higher levels of stress have been reported since the onset of the COVID-19 pandemic^2,3^. Khan et al.^4^ explored the impact of COVID-19 pandemic on the ASD and their caregivers. They utilized a questionnaire to collect data from 58 confirmed ASD persons from a hospital in Doha, Qatar using Revised Overt aggression Scale. The outcome indicated positive influence on autistic individuals in terms of aggregation. However, restrictions put extra pressure on the caregivers and they suggested special considerations from the government for caregivers. In another study, Brondino et al.^5^ investigated challenges imposed by COVID-19 restrictions on autistic persons in Italy. They focused on people with severe ASD in the first two weeks of the restrictions due to the pandemic and concluded that the restriction did not significantly impact the autism people. Nuñez et al.^6^ investigated predictors of behavioral problems in autistic children. They analyzed a cross-sectional data that was collected from 118 autistic children. The results showed raising behavioral problems due to restrictions and they suggested considering the probability of other outbreaks influencing autistic children. Bellomo et al.^7^ studied the reasons of special treatments of children with autism during pandemic COVID-19 and recommended strategies for decreasing stressful factors for autistic children and their parents. As a result, they suggested preparation of online resources and structural changes to help caregivers.

There are research studies specifically focused on the effect of COVID-19 on parents. Vasa et al.^8^ worked on particular types of psychiatric difficulties that appeared a few months after starting of the outbreak and investigated the possibility factors of forecasting changes in these psychiatric symptoms in 257 parents. According the results, it was declared that ASD children and their families need specific psychiatric supports during and after the pandemic. Mutluer et al.^3^ explored the questions of how autistic children reacted to the pandemic and which of their behaviors had altered and how stress of parents is related with these behavioral alterations. In total, 87 children with autism contributed in this study. They found that autistic children had problems to comprehend what is the COVID-19. All subscales of Aberrant Behavior Checklist (ABC) were remarkably dissimilar in pre- and post-pandemic outbreak conditions. Colizzi et al.^9^ aimed to probe whether clinical features or pre COVID-19 socio-demographic could foretell a negative result or determine the demands of the sample consisted of 527 participants Findings demonstrated that as a result of the COVID-19 pandemic some problems appeared in coping with daily life activities, particularly spare-time and structured activities, and also, children showed increased maladaptive behaviors. They revealed that the pandemic has certainly caused increased problems between autistic children. Janessa et al.^2^ surveyed the impact of COVID-19 on stress level of parents had autistic children and amount disruption in their life. They collected data from parents utilizing an online survey in Michigan State, US. The results disclosed that parents or caregivers of autistic children experienced more stresses due to stopping remedial services and the stress level were higher in those parents and caregivers of children with significant severity of ASD aspects. They also reported high level of stress among the caregivers with higher intensity of outside work compared to pre-COVID-19.

Children Behavior Checklist (CBCL) is a checklist, also validated in Iran^10^, to quantify behavioral problems in children. This checklist^11^ is employed in many studied to find syndrome ratings in autistic children^8,12–14^. Arias et al.^14^ examined the CBCL to find potential exploitations to diagnose ASD. They concluded that four syndrome with high scale are more pronounce in ASDs. Pandolfi et al.^15^ evaluated sensitivity of CBCL in two groups of autistic children with emotional and behavioral disorder (EBD) and with ASD. Their results showed that in the case of autistic children CBCL presented proper results but for assessing autistic children with EBD additional data is needed. Lugo-Marin et al.^16^ utilized CBCL and SCL-90-R questionnaires to study effect of COVID-19 pandemic on children and adolescent with ASD. They reported changes in several syndrome in the autistic children and reported that after 2 months of lockdowns some improvement were seen in the young ASD autistic children and stress level in adult ASD were notably decreased. However, the condition for parents and caregivers were different which lockdowns cause stress rise in them. Narzisi^17^ proposed two methods of telemedicine for autistic children to adopt treatments to the emergency conditions during lockdown. The models also included some procedure to support parents. Ueda et al.^12^ disclosed the impact of the lockdown on the quality of life (QOL) of autistic children and their parents. They reported influencing aspects that help them to preserve their quality of life. Children in school and their parents filled QOL and CBCL questionnaires. They found that low QOL is related to mothers’ restricted job flexibility and sleep rhythms of the children. The results also showed that stress level of the parents is related to behaviors of children with neurodevelopmental disorders.

Utilization of artificial intelligence (AI) methods in classification, recognition and treatment of autistic children has been attracted many researcher^18–28^. Ghosh et al.^18^ reviewed articles on the artificial intelligence, machine learning and internet of things in automated systems for the purpose of assisting autistic individuals to cope with everyday life needs and to live independently from others. Using a machine learning tool, Wall et al.^20^ evaluated a common instrument in diagnosis of ASD individuals. They concluded that 7 questions out of 93 questions on Autism Diagnostic Interview-Revised (ADI-R) were sufficient to diagnose ASD with high accuracy. They also validated their machine learning model by investigating two other groups and claimed that nearly 100% of ASDs were accurately recognized by answering only these 7 questions. Thus, the machine learning model aids specialists to diagnose ASD individual in a quick and reliable manner.

Studies on the effects of COVID-19 on autistic children have not addressed clearly the correlation between treatment procedures and on the behavioral problems. According to the reports of the parents and caregivers the behavior of autistic children improved at the early stages of the lockdowns. However, numerous pressures were exerted on the parents. On the other hand, statistical analyses performed on diverse groups of autistic children have not been unified and in some cases contradictory results were reported. Therefore, a profound modeling technique including multiple parameters into account is required to collect effects of all factors into account.

In the present study, we aimed to quantify effects of COVID-19 restrictions and speech treatment approaches during lockdowns on the children with autism spectrum disorders using the neuro-fuzzy system. In this regard, a survey including CBCL questionnaire is prepared using online forms. In total, 87 children with recognized ASD participated in the survey. Comparison of the eight syndromes rating scores between pre-lockdown and post-lockdown periods were performed. Moreover, the influence of speech treatment during restrictions on the level of internalizing and externalizing problems is carefully examined using artificial intelligence methods.

## Method of study

### Participants

In total, the sample of 100 children with confirmed ASD were selected (as diagnosed by third party child psychiatrists) to be examined under the supervision of Zehn-e Ziba Clinic, Tehran, Iran. Among them, 87 children’ parents participated in the survey. The others were not available, refused to participate and in 1 case did not completed the survey in due time. Moreover, for this study informed consent was obtained from all legal guardians of the children. The selected children had been under speech therapy prior to COVID-19 restriction as well and there was also access to CBCL completed forms for these autistic children from pre-lockdown examinations. The baseline CBCL data had been collected between 12 and 1 months before lockdowns. The secondary survey were conducted 15 months after lockdown and took 1 month to be completed. The maximum time gap of the two surveys for the same participant was 27 months and the minimum was 16 months.

### Procedure

All methods were carried out in accordance with relevant guidelines and regulations of and the ethics committee of Zehn-e Ziba Clinic approved the research study. The validated Persian CBCL form as utilized by Tehrani-Doost et al.^10^ was converted to a digitized form using Google Forms. This CBCL-Persian form was also validated by Shahrivar et al.^29^. Detail of translation and validation of the Persian form is accessible in these references. The parents/caregivers of selected autistic children were called through phone and their consent were requested to participate in the study. By the term caregiver, we refer to a person in close contact with the child at least 5 days/week and 4 h/day. The parent and caregivers term may be used interchangeably throughout the report. Since all of the selected participants had participated in the same study before COVID-19 lockdown, they were familiar with how to fill out the forms. After acquiring permissions, a link to the Google Forms was sent to them using social media apps or email. Besides CBCL form, there were other surveys regarding type of treatment procedure during COVID-19 restriction and the satisfaction level of the caregivers from the received services. In particular, three types of treatment were distinguished: telehealth, in-person and public health service. Telehealth was defined as receiving therapy through voice or video call in which the therapist and the autistic children were in direct contact without any other person involved. By in-person we referred to private treatment at the home of autistic child or a place determined by parents. Public health service was defined as clinics, hospitals and other centers devoted to rehabilitation of autistic people. Other information including caregiver’s age, number of siblings and if the caregivers of ASD children had COVID-19 were also collected to find any correlations between data. Statistical analyses were used to find correlations between different factors and outputs and also to find most relevant factors.

## Prediction of child behavior using artificial intelligence (AI)

The applications of artificial intelligence^30^ are referenced in the Introduction for prediction and diagnosis of the ASD level. However, many of the presented models were based on the clinical symptoms and standard checklists and scoring. As many other studies on the ASD responses during lockdown and restrictions of COVID-19 suggest that there are other non-predicted behaviors during such periods for both caregivers and autistic children. The authors believe that with emerge of AI methods in the field of clinical diagnosis, it is beneficial to include the parameter of unpredicted discontinuities such as COVID-19 restrictions into consideration for both caregivers and autistic children.

**Figure 1:**
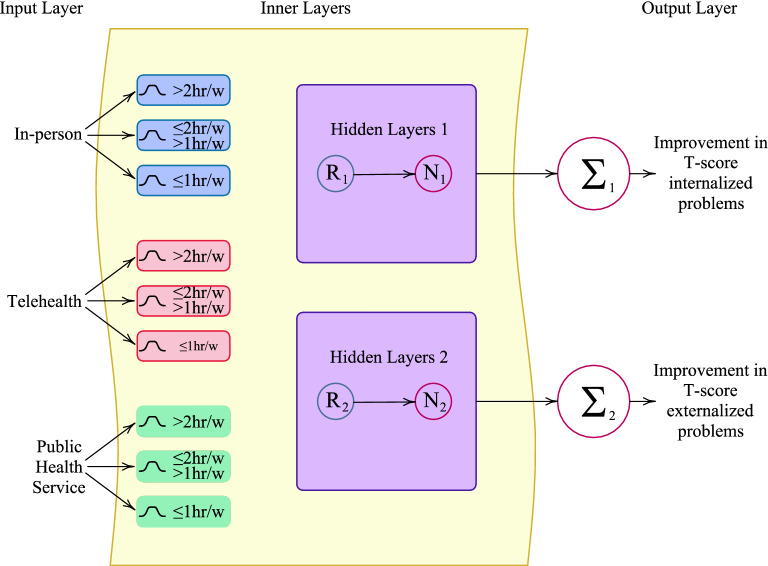
Designed ANFIS network to predict effects of treatment hours per week on the improvement in internalizing and externalizing problems.

Several investigations claim that effects of the lockdowns in different countries were not negative on the ASD persons. However, there are numerous reports revealing severe physical and mental pressure on the caregivers. The influence of the lockdowns on the caregivers are not in the scope of this study and we focus on the AI networks predicting internalizing and externalizing problems in ASDs. Thus, the AI network in Fig. 1 is designed to incorporate the treatment procedures during restriction into prediction of the improvement in T-score of internalized and externalized problems as the result of treatments during restrictions. In this network, two neuro fuzzy systems are utilized having the same input values to predict two distinct outputs. The tools provided in Matlab software (Neuro-fuzzy Designer Toolbox Release 2018b, The MathWorks, Inc., Natick, Massachusetts, United States.)^31^ for neuro-fuzzy systems were utilized to construct neural networks. The advantage of ANFIS is to fuzzification of the input data to linguistic description low, average and high values. For example, the duration of treatment below 1 h per week is considered as low while over 2 h per week is categorized as high. Different rules applies for different categories. At the end of the calculation, a defuzzification procedure converted (mathematically combined) the categorized values into one value of output. The sub-categories of each treatment is illustrated in Fig. 1. Interested readers are referred to^32^ for more details on the calculations and performance of ANFIS networks.

In summary, the treatment hours of autistic children per week were divided into 3 categories of In-person, telehealth and public health service. No-treatment and partial treatments are categorized under partial treatments. Each category has three sections of high (above 2 h/week), medium (between 1 and 2 h/week) and low (below 1 h/week). Some children receive a combination of the treatments each week and during the pandemic. At first, based on the values of each category a membership value is determined. Combinations of the different membership values and initial value of each category gives one of the outputs.

## Results

The demographic information of the participants in the CBCL is summarized in Table 1. The age of the autistic children are between 6 and 13 YoA with the mean age 8.54 and standard deviation 2.04. In addition to the questions in validated CBCL, some questions on the treatments received by autistic children during COVID-19 restrictions were also asked from parents aiming to qualitatively evaluate their level of satisfaction.

**Table 1:** Demographic and clinical data of CBCL participants (n = 87).

Parameter	Value
**Total number, *n***	
–	87
**Age (Mean, SD)**	
–	(8.54, 2.04)
**Gender**	
Male	69 (79.3%)
Female	18 (20.7%)
**Number of siblings**	
1	51 (58.6%)
2	12 (13.8%)
3	20 (23.0%)
Over 3	4 (4.6%)
Missing	0 (0%)
**Parent**	
Mother	79 (90.8%)
Father	8 (9.2%)
Other	0
**COVID-19**	
Parents	27 (31.0%)
Children	11 (12.6%)
**Language level**	
Full sentence	35 (40.2%)
Phrases (2–3 words together)	12 (13.8%)
Single word	24 (27.6%)
No word	16 (18.4%)

The questions on the online form requested some data on the type of speech treatment during restriction and asked them to provide hour/week data for each method and also their feelings about the effectiveness of the treatments. The answers are summarized in Table 2 for different treatments and level of satisfactory as five level Very Much satisfied (= 5), Somewhat satisfied (= 4), Feel neutral (= 3), Somewhat dissatisfied (= 2) and Very much dissatisfied (= 1) in answer to question: How do you feel about the effectiveness of the treatments on your child?” According to Ferguson et al.^33^.

**Table 2:** Feeling of the cargivers about the treatment method and effictiveness.

Satisfaction level	In-person treatment	No treatment	Telehealth	PLS treatment
Number of ADS	23	8	9	47
Very Much satisfied (= 5)	6	0	0	3
Somewhat satisfied (= 4)	10	1	1	11
Feel neutral (= 3)	4	1	6	25
Somewhat dissatisfied (= 2)	3	2	1	7
Very much dissatisfied (= 1)	0	4	1	1
Weighted Mean	3.83	1.88	2.78	3.17

**Figure 2:**
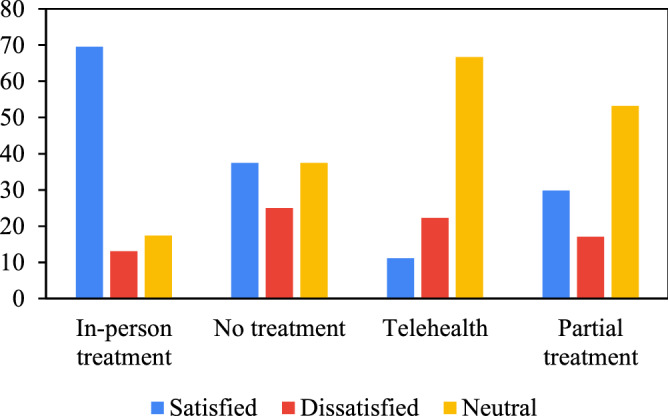
Percent of satisfaction for each speech therapy method provided by caregivers.

The data in Table 2 are summarized in the bar chart in Fig. 2. In this figure, very much satisfied and somewhat satisfied are combined into “satisfied” and somewhat dissatisfied and very much dissatisfied are also combined into “dissatisfied”. As seen, the satisfaction for in-person treatment is far beyond other treatment methods with 69.6% satisfaction level. Telehealth method, although with a limited number of autistic children receiving this treatment, has the minimum satisfaction as reported by caregivers. In autistic children who received no treatment at all, there are also seen satisfactions of the caregivers. On the other hand, partial treatment from public health service seems to have no impact on the satisfaction of the caregivers. From this results it could be deduced that changing therapies for autistic children from telehealth and partial clinical treatment to personal treatment could have more effects at least on the level of satisfaction of the parents. On the other hand, this level of satisfaction is mostly the outcome of change in behavior of their children. In addition, other factors are important. Achieving this satisfaction may also be a result of sparing some extra time for parents to rest and to perform their personal tasks during day which is only possible in the in-person treatments. Other methods require constant care and involvement of parents. Since the number of the participants are comparatively low, the general conclusion may be obtained having a larger group of participants.

**Figure 3:**
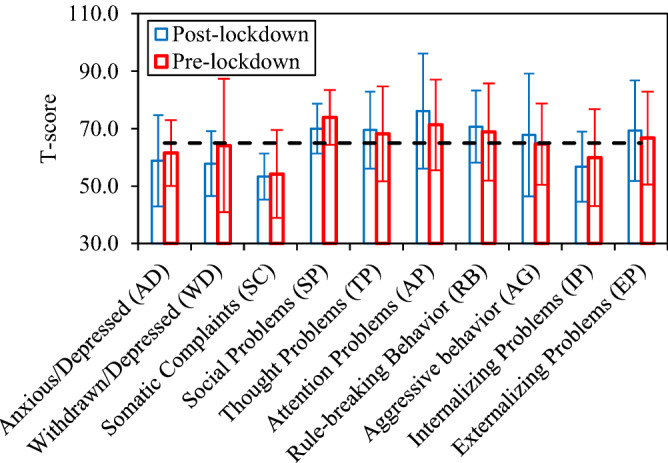
Comparison of T-score ratings of CBCL pre- and post-lockdown.

Figure 3 shows the impact of COVID-19 on the T-score of eight syndrome in CBCL. The formula for calculating T-score is as follows:

1 $$T = \left( {10 \times z} \right) \times 50$$

In which, $z$ represent the z-score in the normal distribution calculated as below:

2 $$z = \frac{x - \mu }{\sigma }$$

In the above formula, $x$ is the obtained value for factor (syndromes) as calculated using collected data of the CBCL questionnaire, $\mu$ and $\sigma$ are the mean value and standard deviation of the same factor of a sample group of the community as presented in validated CBCL studies. Mean T-scores are provided for both pre- and post-lockdown conditions. It is observed that AD, WD and SP syndromes were alleviated during restrictions in autistic children of this study. SC and TP syndromes seem to receive slight influences from restriction. However, lockdowns cause AP, RB and AG syndromes to intensify.

The details of data demonstrated in Fig. 3 are presented in Table 3 with internalizing, externalizing and total problems included. Internalizing problems score is the sum of scores of AD, WD and SC problems scores and externalizing problems score is the sum of RB and AG problems scores. From the ratings of internalizing and externalizing problems it is obvious that COVID-19 restrictions reduce externalizing problems of the ASD children between 6 and 13 YoA and intensifies internalizing problems. The total problems had slight decrease of 1.6 in total T-score. These results is of extreme importance since a meaningful change is observed in the behavior of the autistic children during and in post-lockdown periods. Both positive and negative consequences are seen in these results. The values of standard deviation in some problems are large and makes the mean value of the T-score to be not-conclusive. The large value of the standard deviation indicates the diversity of the results. However, in some problems including SC and SP the low values of standard deviation makes them more reliable. In any condition, keeping these values in mind, the discussion will be on the mean values of the problems.

**Table 3:** Values of T-score ratings of CBCL syndromes for pre- and post-lockdown durations.

	Post-lockdown		Pre-lockdown	
	T1—Mean	SD	T2—Mean	SD
Anxious/depressed (AD)	58.8	15.9	61.5	11.4
Withdrawn/depressed (WD)	57.8	11.3	64.1	23.2
Somatic complaints (SC)	53.3	8.0	54.2	15.3
Social problems (SP)	70.0	8.7	73.9	9.5
Thought problems (TP)	69.5	13.4	68.2	16.5
Attention problems (AP)	76.1	20.0	71.3	15.8
Rule-breaking behavior (RB)	70.7	12.5	68.8	16.9
Aggressive behavior (AG)	67.8	21.4	64.6	14.2
Internalizing problems (IP)	56.7	12.2	59.9	16.9
Externalizing problems (EP)	69.3	17.5	66.7	16.2
Total	65.2	18.5	66.8	17.4

**Figure 4:**
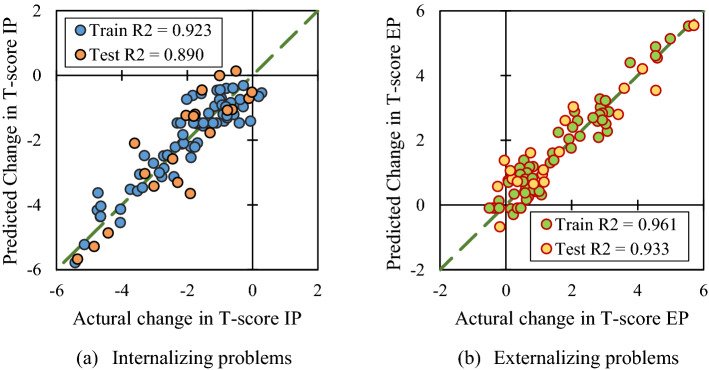
The designed ANFIS network perfomance in predicting changes in T-score due to different speech therapy approaches.

The artificial neural network used 87 dataset which was divided into 67 and 20 single entries to train and test purposes, respectively. In most studies, neural based artificial networks present high accuracy in prediction of the results specifically if the provided dataset has a proper correlation. In the presented network, the performance of the trained model is shown in Fig. 4. The $R^{2}$ value for both train and test data are also included in the graphs. The high values of $R^{2} \ge 0.9$ indicate the high accuracy of the trained model in predicting new speech therapy approaches in the T-score changes in both internalizing and externalizing problems.

The general feature of the graphs shows a noticeable difference between internalizing and externalizing problems changes in terms of T-score. Regardless of the therapy method, during Covid19 pandemic the internalizing problems are alleviated and externalizing problems are intensified.

**Figure 5:**
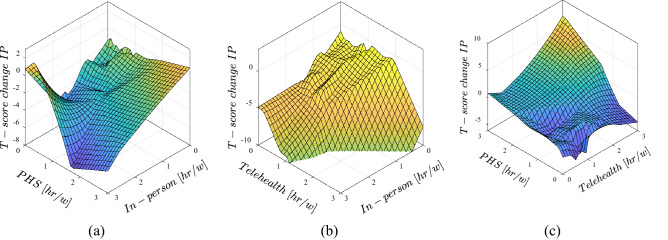
Effects of different speech therapy approaches on the change in internalizing problems in comparison data from pre- and post-lockdown, (**a**) effect of concurrent in-person and PHS treatments, (**b**) effect of concurrent telehealth and in-person treatments, (**c**) effect of concurrent PHS and telehealth treatments.

**Figure 6:**
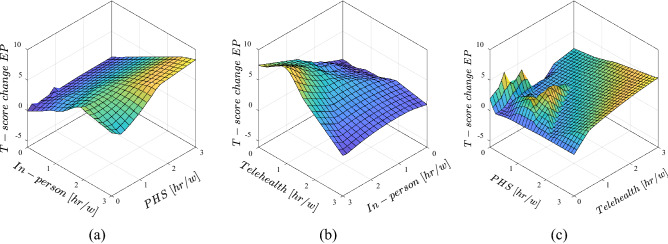
Effects of different speech therapy approaches on the change in externalizing problems in comparison data from pre- and post-lockdown, (**a**) effect of concurrent in-person and PHS treatments, (**b**) effect of concurrent telehealth and in-person treatments, (**c**) effect of concurrent PHS and telehealth treatments.

The effects of different in-person, telehealth and public health service speech treatments on the IPs and EPs are depicted as 3D graphs in Figs. 5 and 6. Effects of different treatment times on the change in T-score of internalizing problems is shown in Fig. 5. It is obvious that the trained ANFIS model suggests that increasing in PHS treatment hours results in decrease in T-score (negative values of T-score change) of IPs as long as the treatment was accompanied by in-person treatment for at least one hour per week (Fig. 5a). However, in lower in-person treatment time and even with telehealth treatment, increase in PHS hours caused negative influence on the internalizing problems. On the other hand, in most scenarios, in-person treatment had a positive impact on IPs (Fig. 5a, b). From the trained model predictions, the effect of telehealth is unclear. While in Fig. 5b it resulted in improvement in IPs, in Fig. 5c it worsened the same problems. Therefore, more data on this treatment method have to be analyzed to reach a reliable conclusion.

The conditions on the externalizing problems are entirely different responses to the treatments. In all scenarios, no changes occur to the EPs’ T-score if there is no treatment at all. Increasing any treatment methods cause an increase in T-score of the EPs. However, some exceptions existed in high hours of in-person treatment. In addition, the telehealth method in high in-person hours reduced EPs significantly. It is worth mentioning, the predictions made by ANFIS model here are performed based on the data available for 87 cases under consideration.

## Discussion

The main objective of the present study was to determine the COVID-19 restrictions on the behavior of ASD children with different speech levels. However, there were some limitations in data collection and in the reliability of the results which are elucidated in the following.

### Limitations of the study

The number of participants in the current study is 87 which is small number for statistical analysis and drawing conclusive results. The reason for this small number of participants is that finding children with CBCL data in a proper time gap (up to 1 year) before COVID-19 lockdown and after 15 months of lockdown was not easy because of elongated time passed before two tests. On the other hand, this small number provides insufficient data for training the ANFIS network which normally requires over 200 sets of data. Moreover, inputs of the neural network are limited to the treatment type and duration. There are several other factor that should be considered. However, the collected data from other factors must be in quantitative manner. However, the ANFIS network utilized in this study could be further extended to include other factors like continuity of treatments and emotional and stress levels of parents. Limitation of telehealth are also one of the obstacles in treatment. Due to increase usage in internet-based applications during COVID-19 pandemic, there are lots of complaint about disrupting internet connections during telehealth session. One another problem is the lack of cooperation from the autistic children with the therapists for different reasons. One of the major reasons is the limited number of tools, similar to toys, used in telehealth.

### Reliability of the study

As mentioned above, the number of the participants in the present study is one of the major limitations. For more reliable results a larger and more diverse groups should be investigated. On the other hand, other checklists like teacher report from (TRF) were not applicable due to limited interactions of teachers with children. The artificial neural network methods present accurate results granted that the input data are accurate and valid enough. In most cases, with increase in the number of datasets finding a pattern of behavior is easier and increases the reliability of the results.

### Comparison to other studies

The main objective of the present study was to determine the COVID-19 restrictions on the behavior of ASD children with different speech levels and effect of the different therapy approaches on their behavioral problems. de Nocker and Toolan^34^ reviewed the articles on the application of the telehealth practices in autistic children therapy. From the results of the current study, it can be seen that almost 50% of the participants received no treatment or insignificant speech treatment. This group of the children demonstrate elevated externalizing problems in comparison to those who receive regular or more treatments. Furthermore, AP and RB rating scores are worsen most likely due to lack of structured environment similar to school, therapy sessions or other environments. Moreover, there are some correlations between language level and externalizing problems as reported by McClintock^35^.

Problems in AD, WD and SC categories alleviated in autistic children after lockdowns. Reduction in social demands could be a possible explanation of reduced WD. This result is in accordance with Lugo-Marin et al.^16^. In addition, a limited social interactions during restrictions results in reduction in AD and SC and in general internalizing problems. However, in Ref.^16^ it is reported that in the initial stages of the lockdowns stress level in both parents and autistic children rose as a result of abrupt change in routine life. In the present study, the survey was performed long after initial days of the lockdown and also stress level in parents was not considered. Moreover, they claimed that in-person treatments could reduce stress and anxiety levels. From the telehealth treatment approach, conclusive results cannot be deduced. Even though autistic children show tendency toward visual instruments, no sign of satisfaction and improvement were seen from the current survey. It must be mentioned that the group of 8 children with telehealth treatment cannot be generalized and more participants with similar conditions are needed for further conclusion. SC problems are a hallmark symptom of ASD and slight changes in the rating of these problems was expected. Improvement in mean total score in autistic children could be regarded as a consequence of decreased environmental requirements and large percent of in-person treatments in the participants of this study.

The neuro-fuzzy system employed in the present study shows proper perspective in prediction IPs and EPs from speech therapy approaches. The trained model predicted that using only one treatment method may cause improvement or deterioration in IPs or EPs. However, from the gathered data influence of combination of two or three concurrent treatments cannot be deduced. The ANFIS model predicts that combination of speech treatment approaches may result in deterioration of the problems even if the treatments individually perform satisfactorily. Thus, using such AI networks could help researchers to construct models that predict some unexpected outcomes from the data.

## Conclusion

In the present study, we quantified the effects of COVID-19 restrictions and speech treatment approaches during lockdowns on the behavior of children with autism spectrum disorders using a neuro-fuzzy system. In this regard, a survey including CBCL questionnaire was prepared using online forms. In total, 87 children with diagnosed ASD participated in the survey. Comparison of the eight syndromes rating scores from pre-lockdown to post-lockdown periods were performed. Moreover, the influence of speech treatment during restrictions on the level of internalizing and externalizing problems was examined using artificial intelligence methods. In performing this study, we encountered several limitations due to COVID-19 restriction to name insufficient number of participants influencing statistical data and reliability of artificial neural network outputs. The main results could be encapsulated in the followings:

- Due to diverse factors affecting behavior of autistic children, exploiting artificial neural network of neuro-fuzzy system is proposed in studying the performance of different treatment approaches.
- It is seen from the collected data of CBCL that restrictions alleviate externalizing problems while intensify internalizing problems.
- Moreover, the collected data and ANFIS analysis suggest that in-person speech therapy is the most effective and satisfactory approach to deal with ASD children during lockdown period.
- Adaptive neuro-fuzzy system provides predictions of externalizing and internalizing problems during restriction as a result of combination of speech treatment approaches.

## Data Availability

The datasets generated and/or analyzed during the current study are not publicly available due restrictions of the institution where the experiments conducted but are available from the corresponding author on reasonable request.
